# Development of practical emergency simulation training in primary health care: Lessons learnt

**DOI:** 10.4102/phcfm.v16i1.4404

**Published:** 2024-05-09

**Authors:** Owen O. Eales, Annelet Kruger

**Affiliations:** 1 Department of Family Medicine, Faculty of Health Sciences, University of Pretoria, Pretoria, South Africa

**Keywords:** simulation training, primary health care, emergencies, drills, clinicians, in-service training, acute care

## Abstract

Emergency care in the primary health care setting is often sub-optimal leading to physician stress and adverse patient outcomes. Formal training opportunities in the management of emergency management are limited in public-sector facilities. Two family physicians conducted emergency simulation drills at primary health care facilities to address this need. The multi-disciplinary team at these facilities were involved, and each drill was followed by reflection and feedback. The drill evolved over an 18 month period, and the simulations as well as the feedback process were reviewed and improved. Reported benefits included improved skills and confidence, teamwork and sharing of information. Staff appreciated the support and the opportunity to review emergency equipment and drugs. Recommendations based on the experience gained and the outcomes of the simulation training include that the feedback is just as important as the simulations and that clinicians need to be trained in order to facilitate simulations successfully.

## Background

Although the focus of primary health care (PHC) is on the management of common conditions, chronic diseases, prevention and health promotion, there is a growing recognition of the importance of emergency care in the PHC setting. In South Africa, the PHC services often have to deal with critical emergencies, overwhelming patient numbers and long delays in transfers to hospitals.^1^

Emergency care in PHC in South Africa is often sub-optimal due to limited skills, training, learning opportunities and equipment.^2^ Clinicians often feel isolated and inadequate when faced with an emergency in PHC. ‘When it come to emergencies in primary care- we are on our own’ (clinician in PHC).^1^ These factors can contribute to adverse patient outcomes in PHC.

The Tshwane District Health Service holds monthly Patients Safety Incidents (PSI) meetings where adverse patient outcomes are discussed. During 2021, these meetings identified gaps in clinician skills in regarding the management of common emergencies PHC facilities.^3^ It became apparent that there is no strategy in the equipping clinicians to deal with emergencies in PHC. The Tshwane District organises regular training on basic life support (BLS) but that is the only compulsory training for clinicians. All other emergency training courses such as Advanced Cardiac Life Support (ACLS) and Advanced Paediatric Life Support (PALS) are optional, and clinicians have to pay for the courses themselves.

Two family physicians decided to use the cases highlighted in the PSI meetings to train clinicians at a community health centre (CHC) using simulation training. Simulation training was chosen as an appropriate platform to train for these emergencies as it offers a controlled environment that gives participants the opportunity to practice skills and also provides immediate feedback and debriefing.^4^ Although simulation training in Africa has unique challenges related to the exorbitant cost of new technologies and manikins, there are also opportunities to develop innovative and relevant training for the African context.^5^

From February 2022 to August 2023, 12 simulation trainings were held in different PHC clinics as part of an outreach programme by family physicians. Topics covered that included approach to chest pain, decreased level of consciousness, convulsions in adults and children, anaphylactic reaction, acute severe asthma, dehydration plus shock in children and cholera.

## Process

The process of planning, conducting and evaluating the simulation training evolved over time from the first training conducted in February 2022. The focus of the initial drills was determined by PSI cases in the district. Over time, a list was developed with the common and important emergencies presenting at primary care facilities with input from clinicians and managers.

For each drill, a simple 10-point scoring system to assess the competency of the group was developed by the family physicians. This was based on the 10 steps or actions deemed as essential during the management of the specific emergency and one point was awarded if the action was included in the resuscitation. Previous experience in conducting simulation training as well as primary care guidelines and BLS principles were used to develop each assessment tool.^6^ A point for teamwork and role allocation was also included.

The first drills took place biweekly at a CHC in Tshwane district. The facility management and clinicians were informed beforehand that a drill would take place but they were not informed of the topic of the drill.

One of the family physicians usually played the role of the patient while the other observed and documented the score and other observations. The simulation was based on a probable patient scenario and took place in the actual clinical workspace in the facility with the clinicians and resources available on that particular day. The intention was to simulate a real resuscitation scenario as closely as possible. This approach also assisted in identifying challenges with equipment and drugs.

The clinicians involved in the simulations varied, with doctors and nurses always present, and at times medical students and interns as well. In general, the clinician who was on duty in the particular service area was asked to attend to the simulated patient and then choose their resus team. The first simulation was typically disorganised with no clear team lead. The call for help was generally omitted and there was rarely a timekeeper, or a scribe allocated to document the resuscitation. Personal protective equipment was also not considered.

A simulation training was followed by feedback following the reflective approach, where the group was asked ‘what did you do well?’ and ‘what can you improve?’ The score was shared and the gaps discussed. On average the teams scored between 5 and 8 out of 10. The next step was to ask a senior medical officer to showcase the same simulation, but now done correctly keeping the feedback in mind this repeat simulation proved to be invaluable, as not only could the staff hear what they were supposed to do, but also see the correct way to do the simulation.

After a few emergency drills in the initial CHC, the family physicians decided to do similar drills during their monthly support visits to outlying clinics with other district-based family physicians.

An important learning point from these subsequent drills was that when a group of family physicians all gave feedback on the simulation, it led to feedback overload. Subsequently, it was decided that only one person should facilitate the feedback, and the focus should be on positive and constructive feedback while avoiding criticising the clinicians.

After attending the Emergency Triage and Treatment (ETAT) course for children, the family physicians also initiated paediatric simulations using a paediatric simulation doll.

Both family physicians attended the Training the Clinical Trainer (TCT) course and realised that the simulation training is not only useful for teaching skills but can also be used to train trainers. They decided that different people should facilitate the training and the feedback, followed by a debriefing session where the facilitator was given feedback on their facilitation of the simulation.

During the cholera outbreak in Tshwane in 2023 facility preparedness for cholera was a big concern. Specific clinics were identified where a cholera case with severe dehydration was simulated. The rubric for this case was shared widely to all medical officers in Tshwane District, and simulations were done in various clinics as preparedness for cholera.^7^

## Outcomes

- All clinicians involved showed improved levels of skills.
- There was dissemination of information and resources such as apps.
- Improved levels of confidence were reported and demonstrated.
- Equipment and emergency drug shortages were identified.
- Clinicians worked better together as a team.
- Staff felt supported.
- Opportunity arose to discuss other emergency cases.

**FIGURE 1:**
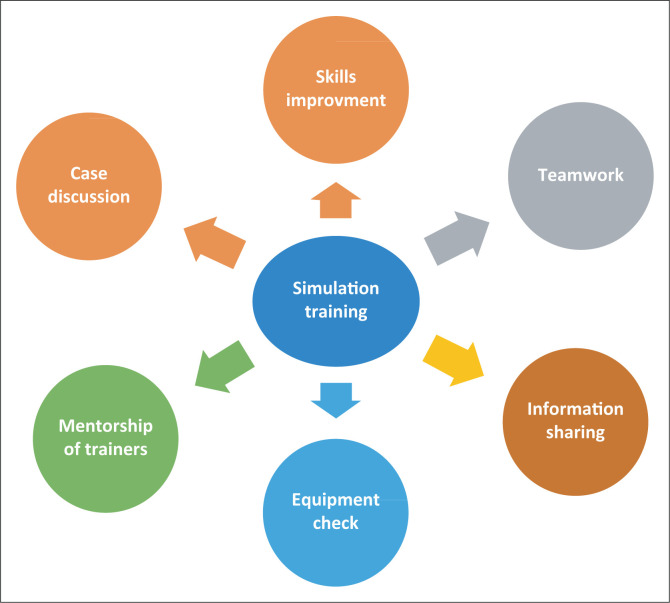
Outcomes of simulation training.

Our findings echo the findings of Weersink et al.^8^ that found multiple advantages to simulation training (Figure 1).

## Recommendations

- You need a simple rubric to evaluate the simulation.
- Let the group evaluate themselves before the facilitator evaluates the group.
- Feedback should start with the positives and transition into what improvements need to be made.
- Do not let too many people give feedback.
- Simulation training is an opportunity to mentor trainers.
- Simulation training should not only focus on the clinical aspects but also on teamwork and role allocation.
- Various electronic applications are valuable, for example, Pedihelp, GTFCC cholera and EM guidance.^9^
- In order for simulation training to be adopted on a wider scale, clinicians need specific training on how to facilitate this training.

In conclusion, this case study shows our learning experience of training clinicians in the workplace, by using existing infrastructure and equipment, in a simulated situation as close as possible to real life.
